# The comparative perspective of phytochemistry and biological properties of the Apiaceae family plants

**DOI:** 10.1038/s41598-023-39254-8

**Published:** 2023-07-31

**Authors:** Mohammad Hamidian, Amin Salehi, Reza Naghiha, Mohsen Movahhedi Dehnavi, Ines Castangia, Maryamossadat Nejad Mirfathi

**Affiliations:** 1 Department of Agronomy and Plant Breeding, Faculty of Agriculture, Yasouj University, Yasouj, Iran; 2 Department of Animal Sciences, Faculty of Agriculture, Yasouj University, Yasouj, Iran; 3 Department of Life and Environmental Sciences, University of Cagliari, Cagliari, Italy; 4 Department of Chemistry, Yasouj University, Yasouj, Iran

**Keywords:** Antimicrobials, Biochemistry, Antimicrobials

## Abstract

Despite the availability of numerous reports on the discovery of medicinal plant compounds and their properties, one may encounter contradictory results released by these reports at the level of plant families and even within species. To establish an accurate perspective of the Apiaceae family, this study examined the fruit essential oil and methanolic extract of wild and common species of this family. According to the measurement of the antioxidant property in the methanolic extract of the fruits using the 2,2-diphenyl-1-picrylhydrazyl (DPPH) method, *Ferula gummosa*, *Pimpinella anisum* and *Cuminum cyminum* have high power in inhibiting free radicals. However, *Bunium persicum* had the strongest DPPH radicals inhibitory potential among all essential oils. The results of antimicrobial tests and their classification analysis showed that *C. cyminum* and *B. persicum* fruit essential oil with a high amount of cuminaldehyde had the most antibacterial properties. At the same time, the antifungal properties of *H. persicum* essential oil (rich in aliphatic ester) were stronger than those of the all the studied plants. Also, the essential oils of *F. gummosa* and *Kelussia odoratissima* had favourable antimicrobial properties compared to other studied plants. The investigation of the bacterial structure by scanning electron microscope confirmed the effect of the applied essential oils dose and their antibacterial potential. In general, for the first time, this paper determined the biological values of the fruit essential oil of some wild plants, such as *K. odoratissima* and *H. persicum*. Besides, in vitro examination and the mathematical models provided a suitable classification, which makes a comprehensive view in terms of the properties of the Apiaceae family.

## Introduction

The improper and uncontrolled use of antibiotics and the high ability of bacteria to adapt to adverse environmental conditions have led to the emergence of multidrug-resistant strains in bacteria, which is a significant threat to public health^1^. In the same way, resistance to various fungicides in many pathogenic strains of fungi is a similar consequence of the uncontrolled use of chemical antimicrobials^2^. The promising results of herbs as natural sources in pharmaceutical and food industries, along with the increasing public interest in herbal medicines, have led many researchers in various scientific and applied fields to measure the composition of each plant and the applied fields of their metabolites^3^. Hence, ethnobotanical and phytochemical investigations are essential in detecting secondary medicinal plant components to determine their biological properties. Essential oils, as rich sources of plant secondary metabolites, have antioxidant, insecticidal, fungicidal, bactericidal, and even herbicidal properties. To this end, the application of these compounds has increased in various industries^4^.

Due to the hydrophobic nature of essential oils, these compounds cause disruption and destruction in the cell membrane of fungi, yeasts, and bacteria, followed by considerable cellular damage and, eventually, cell death in these microorganisms. However, the intensity of these properties largely depends on the chemical profile of each essential oil^5,6^. The family of Apiaceae Lindl. includes economically important vegetables, herbs, and spices, where it is considered one of the most highly frequent plant families with well-known antioxidant, antibacterial, and antifungal properties^7^. In different organs of plants (e.g., fruits, aerial organs, and roots) of this family, there are schizogenous secretory canals and ducts for essential oils that store these valuable metabolites with different qualities and quantities in each organ^8^. Iran is an important country in the world regarding the abundance of species of the Apiaceae family, and is ranked third after China and Turkey^9^. The properties of many endemic and wild plants of this family in Iran are unknown throughout the world. Therefore, in addition to the ecological and genetic values of these plants, the identification of compounds, medicinal, and antioxidant properties of these plants also assumes crucial importance.

As it has been mentioned, the essence of Apiaceae family plants contains a wide range of compounds with varying biological properties. *K. odoratissima* Mozaff. is an endemic and endangered medicinal plant available in the flora of Iran, which is used as a traditional medicinal and spice plant (particularly in distribution areas)^10^. A major part of the essence of *K. odoratissima* plant consists of (*Z*)-ligustilide (phthalide) and germacrene D (monocyclic sesquiterpene). In addition to its traditional uses as a fresh and processed vegetable in dairy products, this plant is used as a medicine for the treatment of rheumatic disorders, blood pressure, indigestion, inflammation, and antispasmodic pains as well as the treatment of digestive problems, due to its compounds^11^. On the other hand, many properties and applications have been discovered in some more well-known species of the family plant. These plants have different uses based on the compounds existing in the fruit essence. For example, cuminaldehyde, as the main compound of *Cuminum cyminum* L, has commercial applications in the perfumery and cosmetics industry, especially as an antifungal, antibacterial and antioxidant agent^12^. *Foeniculum vulgare* and *Pimpinella anisum* fruit oil (rich in anethole) are consumed in moisturizing cream formulas, skin creams, lotions, ointments, other cosmetics, and personal care products. Furthermore, it is used as a perfumery agent for oral care products^13^. Carvone is one of the other compounds found in the essence of *Anethum graveolens* plant. This compound has anti-cholesterol and antioxidant activity in medicine, insecticidal properties for pest control, and an inhibitory effect on the growth of potato tubers. It has been proven its usability for potatoes in the post-harvest storage and transportation stages^14^. *F. gummosa* Boiss is another medicinal plant of the Apiaceae family of the genus *Ferula* L. that grows in southwest Iran and the foothills of Zagros Mountains^15^. Oleo-gum-resin contains valuable monoterpene secondary metabolites and its beneficial applications have been proven in various medicinal items such as laxative, expectorant, anti-nociceptive, antiseptic, anti-hysteric, and Anti-catarrh items, besides its use in glue manufacturing for industrial and cosmetics consumption^16^. *K. odoratissima* and *F. gummosa* are generally less known, and there are few reports about their essential oil properties, while these species are at risk of extinction in Iran. Besides them, *Heracleum persicum* Desf. (genus *Heracleum*) is another plant of the Apiaceae family. Unfortunately, there are limited studies pertaining to its antioxidant and antimicrobial activity. *H. persicum* is an annual Iranian plant that grows in humid and mountainous areas with an altitude of more than 1500 m in the north, northwest, center, and northeast of Iran, especially in the provinces of Mazandaran, Azerbaijan, Khorasan, Tehran, Yazd and Kerman^17,18^. It is normally used as a flavouring and spicy in traditional culture, while it is also used in folk Iranian medicine as a treatment for diarrhoea, indigestion, flatulence, infection, and vomiting constipation. Some studies have reported anticonvulsant properties for this plant^19–21^.

Although studies on some well-known species of the Apiaceae family have begun years ago, there are still shortages or contradictions between these reports. For example, prior reports have focused on the essential oil of the aerial part of these plants. In comparison, the fruit of many of these plants and their essential oil have consumption for spice, folk medicine, alcoholic beverages, flavourings, and perfumes. On the other hand, the high level of contradiction in reports on the essential oil properties of the Apiaceae family does not provide clear insight into each plant's potential compared to other family plants. For instance, Oroojalian et al.^22^ reported that the range of minimum inhibitory concentration (MIC) was about 7.5 and 3 mg/mL in *Cuminum cyminum* essential oil for bacteria such as *Staphylococcus aureus* and *Escherichia coli*. Whereas, other reports about the essential oil of this plant showed that MIC was about 25 and 50 µg/mL in *S. aureus* and *E. coli* bacteria^23^. These mentioned distinctions have existed in other plants related to this family. For example, regarding *Foeniculum vulgare*, the MIC range of fruit essential oil against *S. aureus* and *E. coli* bacteria has been reported equal to 250 and 125 µg/mL^24^. However, in another research, MIC was > 10.0 and 0.25 mg/mL against the same bacteria^25^. Alternatively, about *Anethum graveolens* fruit essential oil, the MIC range against *S. aureus* and *E. coli* ranged from > 10.0 mg/mL to 5 µg/mL in different studies^26,27^. Although the compositions of essential oils and bacteria strains may be different in these studies, the striking differences between mg/mL and µg/mL in inhibitory concentrations make it very hard to select and classify plants according to their specific properties, especially in intra-familial classes. In addition, these distinctions could be found in many reports concerning antifungal and antioxidant properties. Hence, the absence of any clear vision of the wild and common species of these plants makes an unreliable condition and prevents the expansion in the practical and scientific fields. Therefore, the purpose of the present study is to investigate some biological properties of the nine species of the Apiaceae family, considering the results of prior studies on the studied species. In this way, a comprehensive study of these species can lead to a summary of the properties of each plant species based on the chemical profile of their essential oils. In the same way, in order to accurately compare the effect of the essential oils of the studied plants on the destruction levels of bacteria, one gram-positive and one gram-negative bacterium were selected and the effects of these essential oils on the two bacteria were examined by Scanning Electron Microscope.

## Material and methods

### Materials

In the present study, the materials included Mueller hinton agar (MHA) (Merck), Mueller hinton broth (MHB) (HIMEDIA), Sabouraud dextrose agar (SDA) (QUELAB), Sabouraud dextrose broth (SDB) from (QUELAB), Gentamicin (10 µg per disc) and Ciprofloxacin (5 µg per disc) antibiotic disc diffusion (Padtanteb, Iran), Voriconazole Antibiotic disk diffusion (1 µg per disk) (RIDACOM). These materials were all purchased; moreover, all chemicals except 2,2-diphenyl-1-picrylhydrazyl (DPPH) (Sigma Aldrich) were purchased from Merck.

### Plant material

Wild plant material was collected with the consent of the Natural Resources and Watershed Management Organization. Since some species are widely distributed and cultivated throughout the country, no legal requirements or permission was required to gather the samples. The collection of the plant material complied with the relevant Natural Resources and Watershed Management Organization and national and international guidelines and legislation. *F. gummosa* fruits (31° 01′ 29″ N 51° 21′ 53″ E) were freshly collected from Sisakht, Kohgiluyeh and Boyer-Ahmad Province, *K. odoratissima* fruits from the Khuyeh region in Chaharmahal and Bakhtiari Province (32° 41′ 11″ N 49° 49′ 42″ E), and *H. persicum* from Khalkhal, Ardabil, Iran during the fruiting period in August 2021. The exact species of *F. gummosa* (herbarium number: PM1393), *K. odoratissima* (herbarium number: PM1396) and *H. persicum* (Herbarium number: PM1392) were determined in the Herbarium of Pharmacology Department, Shiraz University of Medical Sciences, Shiraz, Iran. The fresh fruit of *F. vulgare* (var. azoricum), *Petroselinum crispum* (var. Neapolitanum) and *A. graveolens* was prepared from Pakan Bazr Company, Isfahan. Similarly, Fresh fruits of *C. cyminum* and *P. anisum* were purchased from Razavi Seed and Seedling Institute (Razavi Khorasan province) and *B. persicum* was prepared from Agricultural and Natural Resources Research Center of the Kerman province. The fruits of all the plants were dried (in the shade at room temperature). Then, the essential oil and methanol extracts were extracted and the samples were stored at 4 °C in the refrigerator.

### Methanolic and EOs extraction of fruits

The essential oil of each plant was extracted several times using distillation with water by the Clevenger apparatus. The essential oils of each plant were stored at 4 °C in a dark and closed container in a dark place until use. The methanolic extract was obtained from the fruit (9 g) by a Soxhlet extractor with methanol solvent. Finally, the extract was purified by a rotary evaporator. To make the mother methanolic extract based on the amount of fruits, all the pure extract was again diluted with methanol to 30 mL (300 mg fruit/mL) and stored at 4 °C in dark and sealed containers. At the time of measuring the antioxidant properties, different concentrations (100, 50, 25, 12.5, 6.25 and 3.125 mg fruit/mL) of the main methanolic extract were prepared based on the amount of fruit extracted from the final solution volume.

### Total phenolic and flavonoid content

The test solution for estimating the total phenolic content of each fruit contained methanolic extract and Folin–Ciocalteu reagent to which sodium bicarbonate was added after placing in Bain-marie. The test solution for estimating total flavonoid content also contained aluminum chloride, methanolic extract, potassium acetate, and distilled water that was placed in Bain-marie at 25 °C. Finally, the total phenolic content was measured according to the absorbance of the test solution at a wavelength of 760 nm and based on the absorbance of standard concentrations of gallic acid equivalent (GAE)^28^. The total flavonoid content was also measured based on the absorbance of test solutions at 415 nm using a spectrophotometer (Lambda 210 EZ) according to the absorption of standard concentrations of quercetin (quercetin equivalent or QE)^29^. The results are reported based on the mean of 3 replications with ± standard error.

### DPPH radical scavenging activity

The antioxidant power of extracts and essential oils was reported based on the concentrations of the extract or essential oil scavenging 50% of DPPH radicals (IC_50_). To this end, 1 mL of different concentrations of the sample was added to 0.25 mL of 0.2 mM DPPH solution. Accordingly, the absorbance of each sample was read at 517 nm using the spectrophotometer after 30 min of rest at room temperature and in the dark^30^. The results were reported based on means of 3 replications with ± standard error.

### Reducing power

To measure the reducing power, 2.5 mL of K_3_Fe(CN)_6_ 10%, 2.5 mL of 0.2 M potassium phosphate with pH 6.6, and 1 ml of different concentrations of the sample were mixed. In addition, 2.5 mL of Trichloroacetic acid (10%) was added to the resulting mixture after its placement in Bain-marie at 50 °C for 25 min and was then centrifuged. Finally, the test solution containing 2.5 ml of distilled water, 0.5 mL of 0.1 ferric chloride, and 2.5 mL of the centrifuged mixture was read by the spectrophotometer. The final report was based on a concentration of the samples and was equal to 0.5 at a wavelength absorption of 700 nm (EC_50_)^31^.

### Analysis of the essential oil

Gas chromatography–mass spectrometry (GC–MS) analyses were performed with an Agilent 7890 A gas chromatograph equipped with a HP-5MS fused silica column (30 m × 250 μm coated with 5% phenyl methyl silicone, 95% dimethylpolysiloxane, 0.25 μm film thickness), interfaced with an Agilent mass selective detector 5975C inter MSD. Oven temperature was programmed to rise from 60 to 220 °C at a rate of 3 °C/min; and the transfer line temperature was 260 °C. The carrier gas was Helium with a flow rate of 30.6 cm/s and a split ratio of 1:100. Mass scan range from 40 to 300 a.m.u. The identification of oil components was assigned according to the retention index of each GC–MS compound. For measuring the content of each compound, the spectra were considered to be simultaneous if the similarity index was high (%95%). The Kovats index (KI) was also calculated according to compatible Kovats indices (KI) on databases (WILEY, NIST, and PHEROBASE).

### Microbial strains

Antimicrobial properties were measured on 4 bacteria and 2 fungi (prepared from the Persian Type Culture Collection). The bacteria included *Pseudomonas aeruginosa* (ATCC 9027), *Escherichia coli* (ATCC 25922), *Bacillus subtilis* (ATCC 6633), and *Staphylococcus aureus* (ATCC 6538). The fungi included *Aspergillus flavus* (ATCC 15548) and *Candida albicans*. It is noteworthy that the bacterial strains were stored at − 85 °C (in 20% glycerol).

### Agar diffusion test

This procedure was performed according to the instructions of document M51-A2 of the Clinical Laboratory Standard Institute^32^. Bacterial and fungal suspensions were cultured in Petri dishes containing sterile food media to evaluate the antimicrobial properties based on disk Agar diffusion. It should be noted that Mueller hinton agar (MHA) and Sabouraud dextrose agar (SDA) were used for bacteria and fungi, respectively. The suspensions of microorganisms were prepared freshly and on a daily basis for the tests. The sterile Mueller Hinton Broth (MHB) medium was used for bacteria and Sabouraud dextrose broth (SDB) for fungi. Incubation conditions for bacterial suspension included 37 °C for 24 h. Its concentration was based on 0.5 McFarland Turbidity Standard. In preparing the fungus suspension, it was first prepared in a petri dish containing 7-day SDA Spore Bank. The suspension was then prepared by sterile 0.85% saline solution from the spore bank (~ 10^5^ spore. mL^−1^), and 15 µL of EOs were added to the blank disks in each petri dish. For the better diffusion of the biomolecules of the essential oil, each petri dish was first exposed to 4 °C for 4 h. The incubation was then performed on bacterial samples at 37 °C for 24 h, yeast samples at 28 °C for 48 h, and fungal samples at 28 °C for 72 h. The antimicrobial activity of each essential oil was determined by a caliper based on inhibition zone (mm) measurements. The results were reported based on means of 3 replications with ± standard error. The controls of ciprofloxacin (5 μg per disc) and genatamicin (10 μg per disc) of Padtanteb, Iran were used for bacteria; and the Voriconazole (1 μg per disc) of RIDACOM brand was used for fungi and yeast.

### Minimum inhibitory concentration (MIC), minimum bactericidal and fungicidal concentration test (MBC/MFC)

Owing to the liquid nature and different essential oils, the density of each one was separately determined to calculate and report different concentrations in mg/mL. MIC and MBC/MFC were estimated according to the broth microdilution method^33^. The content of each well of the 96-well micro-plates included 100 µL of different dilutions of essential oils (EOs), 95 µL of liquid culture medium (MHB for bacteria and SDB for fungi), and 5 µL suspension of bacteria and fungi. Different amounts of essential oil concentrations were prepared in 1% dimethyl sulfoxide (DMSO). The incubation conditions were 24 h and 37 °C for bacteria, 48 h and 28 °C for yeast, and 72 h and 28 °C for fungi. The MIC was estimated based on the concentration of essential oil that prevented the growth of bacteria or fungi. In fact, MBC/MFC was the minimum concentration of the essential oil preventing the growth of bacteria or fungi in the re-culture of MIC-related microplate wells in solid culture media.

### Scanning electron microscope (SEM)

Overnight cultures of a gram-positive bacterium, *B. subtilis*, and a gram-negative bacterium, *E. coli*, were adjusted to McFarland 1 standard and then treated with EO constituents at the determined MIC/4 values. After a 1 h incubation period (37 °C) for each bacterium, the cells were harvested by centrifugation, washed five times PBS, and resuspended in sterile distilled PBS. 20 μL of suspension was spread onto a microscope slide and fixed in 3% glutaraldehyde for 2 h. The samples were then diluted in 20% to 100% dilutions in ethanol alcohol solutions. Finally, the slides containing the sample were coated with gold and evaluated using an LEO electron microscope, model 1455VP (Germany).

### Statistical analysis

Principal Component Analysis (PCA) and Agglomerative Hierarchical Clustering (AHC) were analyzed by XLSTAT (2021 version). Statistical analyses were conducted utilizing the SAS version. 9.1. LSD test was used to compare the means.

### Ethical approval

The study was approved by the Institutional Ethics Review Board of the Yasouj university (2021-02-02).

### Human and animal participants

This article does not contain any studies with human or animal subjects.

## Results and discussion

### Secondary metabolite and antioxidant activity of fruits

The amount of total phenol and flavonoids in the extracts of the studied species showed a significant difference (Table 1). The antioxidant properties of methanolic extracts were determined using DPPH and reducing power methods. In many studies, the amounts of antioxidant properties were reported based on the pure solid extract obtained by various solvents that were then purified. In the present study, the measurement of antioxidant properties was based on milligrams (mg) of the extracted fruit since the plants were from different species. Thus, different weights of the solid extract were obtained in the extraction of each species based on a certain amount of the fruit. Therefore, determining the level of antioxidant activity solely based on the weight of the pure extract (solid extract) may not be a reasonable basis for determining the level of the antioxidant activity of the fruit in a comparative study. This is so because a particular pure plant extract may have considerable antioxidant levels. However, the weight ratio of the pure extract per mg of fruit and its antioxidant activity based on fruit weight can be lower than those of other plants. Also, since fruits of all species are used in powder as spices and traditional medicines, the comparison of antioxidant activity in fruits compared to pure extract can be more functional in the present study.

**Table 1:** Variance analysis and mean comparisons of total phenolic compounds (TPC), total flavonoid compounds (TFC) and antioxidant activity of *Ferula gummosa* (FG)*, Petroselinum crispum* (PC)*, Anethum graveolens* (AG)*, Cuminum Cyminum* (CC)*, Bunium persicum* (BP), *Heracleum persicum* (HP)*, Pimpinella anisum* (PA)*, Kelussia odoratissima* (KO) and *Foeniculum vulgare* (FV) fruits methanolic extract.

	FG	PC	AG	CC	BP	HP	PA	KO	FV	Quercetin (µg/mL)	BHA (µg/mL)
TPC (mg GAE/g DW)	21.2 ± 0.07^a^	4.23 ± 0.15^e^	4.17 ± 0.4^e^	5.41 ± 0.23^d^	1.92 ± 0.02f.	9.58 ± 0.47^b^	7.07 ± 0.14^c^	6.05 ± 0.05^d^	6.11 ± 0.23^d^	–	–
TFC (mg QE/g DW)	6.18 ± 0.18^a^	3.33 ± 0.09^d^	1.36 ± 0.02^ g^	3.71 ± 0.065^c^	1.08 ± 0.09^ h^	2.82 ± 0.18^e^	4.73 ± 0.11^b^	2.04 ± 0.13f.	3.37 ± 0.12^d^	–	–
Reducing power EC_50_ (mg/mL)	4.91 ± 0.12^ h^	15.1 ± 0.09^d^	12.22 ± 0.16^e^	7.92 ± 0.15f.	20.83 ± 0.19^a^	5.06 ± 0.46^ h^	6.55 ± 0.62^ g^	17.36 ± 0.36^c^	19.23 ± 0.22^b^	47.55 ± 0.08	52.01 ± 0.1
DPPH IC_50_ (mg/mL)	8.85 ± 0.04^i^	22.2 ± 0.13^d^	27.12 ± 0.49^c^	9.48 ± 0.33^ h^	39.99 ± 0.31^a^	15.82 ± 0.93^e^	10.84 ± 0.13^ g^	34.49 ± 0.08^b^	13.91 ± 0.18^f^.	98.04 ± 0.02	27.23 ± 0.02
	TPC			TFC			Reducing power			DPPH	
Species	**			**			**			**	

Mean ± standard error (n = 3). Similar letters show non-significance based on LSD (p = 0.01).

**Significant at 1% probability levels, respectively.

The amount of TFC and TPC levels in *P. crispum*, *A. graveolens*, and *K. odoratissima* fruits were estimated to be about 4.23 and 3.33 mg/mL, 4.17 and 1.36 mg/mL, and 6.05 and 2.04 mg/mL, respectively (Table 1). The antioxidant power of *A. grav*e*olens* fruit based on the reducing power method with EC_50_ of 12.22 mg/mL was intermediate among all species. The antioxidant activity of *K. odoratissima* fruit was significantly lower than those of *P. anisum*, *H. persicum*, *C. cyminum*, *A. graveolens* and, *F. gummosa* fruits. Generally, the fruit of *F. gummosa* had the highest total phenol content (TPC) (21.23 mg/g) and total flavonoid content (TFC) (6.18 mg/g) and *B. persicum* had the lowest amount of phenolic and flavonoid compounds among others. Since the highest concentration of *B. persicum* was consumed to inhibit 50% of DPPH (IC_50_) and reduce ferric ions (Fe3 +) to ferrous ions (Fe2 +) (reducing power EC_50_), the fruit of this plant had the lowest antioxidant activity. The antioxidant activity of methanolic extract of *F. gummosa* was higher than those of all species. In *H. persicum*, *P. anisum*, and *C. cyminum*, TPC were 9.58, 7.07, and 5.41 mg/g and TFC were 2.82, 4.73, and 3.71 mg/g respectively (Table 1). In general, the second highest amount of TPC and TFC was observed in *H. persicum* and *P. anisum* fruits, respectively. In the same way, *H. persicum* had the second highest reducing power (EC_50_: 5.06 mg/mL). After *F. gummosa* fruit, *P. anisum* had the best efficacy in scavenging DPPH radicals with IC_50_ of 10.84 mg/mL (Table 1). Previous studies have demonstrated that levels of phenolic and flavonoid compounds in a plant species are directly and strongly associated with antioxidant powers such as reducing power and DPPH radical scavenging^34,35^. Therefore, the higher levels of TPC and TFC in *F. gummosa* and *P. anisum* fruit compared to the other plants were the main reasons for its antioxidant power. On the other hand, *C. cyminum* contains a smaller amount of TPC and TFC than some studied species; however, the fruit of this plant had the highest antioxidant activity against DPPH radicals with IC_50_ of 9.48 mg/mL (Table 1). These results can be attributed to the differences in the types of flavonoid and phenolic compounds of the different plants (Table 1). Different phenolic and flavonoid compounds have different antioxidant powers^36,37^.

### Chemical composition and density of essential oils

According to Tables 2 and 3, some differences were revealed in the chemical profiles of essential oils extracted from all the fruits of the plants. The highest range of compounds was identified in *K. odoratissima* (31 compounds), and the smallest one was related to *P. anisum* (8 compounds). On the other hand, Table 4 shows that the density of each essential oil is different due to its unique chemical profile. The chemical type of *F. gummosa*, *P. crispum* and *A. graveolens* essential oils were mostly monoterpene hydrocarbons and oxygenated monoterpenes (Table 2). Also, the density ratios of these plants were about 0.859, 0.826 and 0.873 mg/µL, respectively (Table 4).

**Table 2:** Chemical compounds of *Ferula gummosa* (FG)*, Petroselinum crispum* (PC)*, Anethum graveolens* (AG)*, Cuminum Cyminum* (CC) and *Bunium persicum* (BP) fruit essential oil.

Compounds name	RT (min)^a^	FG (%)^b^	PC (%)^b^	AG (%)^b^	CC (%)^b^	BP (%)^b^	RI^c^
α-Thujene	8.39	0.63	0.52	–	0.41	0.19	930
α-Pinene	8.7	**22.83**	**45.23**	–	1.88	**2.68**	938
Camphene	9.31	0.23	0.31	–	0.16	–	954
Sabinene	9.98	0.68	1.11	–	–	–	973
β-Pinene	10.36	**48.36**	**29.7**	–	**23.35**	**3.61**	980
Myrcene	10.44	2.03	1.31	0.17	1.34	0.77	984
α-Phellandrene	11.23	–	0.23	0.82	0.48	–	1010
δ-3-Carene	11.37	**10.69**	–	–	–	–	1014
α-Terpinene	23.44	–	–	–	0.34	–	1020
*p*-Cymene	11.97	0.43	0.22	0.34	**8.65**	**15.12**	1029
Limonene	12.26	1.96	1.46	**33.12**	0.5	6.19	1034
1,8-Cineole	12.28	–	**4.92**	–	0.33	0.26	1038
*Z-*β-Ocimene	12.34	–	–	–	0.22	0.17	1040
γ-Terpinene	13.28	–	0.47	0.13	**23.01**	**19.49**	1063
Terpinolene	14.47	0.33	0.26	–	0.1	0.38	1089
*trans*-Pinocarveol	16.96	0.98	–	–	–	–	1139
Camphor	17.25	–	–	–	–	0.25	1145
Pinocarvone	18.19	0.38	–	–	–	–	1161
Borneol	18.32	–	–	–	–	0.31	1164
Terpinen-4-ol	18.66	0.59	–	–	0.47	0.91	1175
*p*-Cymen-8-ol	18.9	0.44	–	–	–	0.22	1180
α-Terpineol	19.27	–	–	–	–	0.93	1190
Myrtenol	19.31	1.05	–	–	0.72	0.2	1200
*cis*-Dihydro carvone	19.45	–	–	**3.73**	–	–	1203
*trans*-Dihydro carvone	19.75	–	–	**2.01**	–	–	1221
*endo*-Fenchyl acetate	20.18	**3.73**	–	–	–	–	1254
Cumin aldehyde	21.56	–	–	–	**13.92**	**41.84**	1250
Carvone	21.76	–	–	**54.3**	–	–	1291
(*E*)-Anethole	23.34	–	–	0.1	–	–	1290
α-Terpinen-7-al	23.46	–	–	–	**8.27**	1.48	1290
γ-Terpinen-7-al	23.49	–	–	0.2	**15.34**	**3.32**	1295
δ-Elemene	25.68	0.3	–	–	–	–	1339
β-Cubebene	27.76	0.25	–	–	–	–	1385
β-Barbatene	28.8	0.57	–	–	–	–	1440
(*E*)-β-Farnesene	29.95	0	0.33	–	0.21	–	1451
γ-Muurolene	30.17	0.48	–	–	–	–	1475
β-Selinene	31.68	0.26	–	–	–	–	1496
β-Dihydro agarofuran	32.24	0.35	–	–	–	–	1506
β-Bisabolene	32.39	0.47	–	–	–	–	1509
γ-Cadinene	32.57	0.27	–	–	–	–	1513
Myristicin	32.88	–	**9.34**	0.1	–	0.51	1520
Elemicin	33.77	–	0.86	–	–	–	1540
Carotol	35.23	–	0.57	–	–	–	1571
Guaiol	35.79	0.36	–	–	–	–	1597
6-Methoxy-elemicin	36.1	–	0.33	–	–	–	1607
Dill apiole	36.66	–	–	**4.04**	–	–	1623
β-Eudesmol	38.05	0.58	–	–	–	–	1653
Apiole	38.63	–	0.69	–	–	–	1677
Aldehyde		0	0	0.2	29.26	45.16	
Monoterpene hydrocarbons		88.17	80.82	34.58	60.44	48.6	
Oxygenated monoterpenoids		7.52	4.92	60.04	9.79	4.56	
Oxygenated sesquiterpenoids		0.94	0.57	0	0	0	
Phenylpropanoids		0	11.22	4.24	0	0.51	
Sesquiterpene hydrocarbons		2.6	0.33	0	0.21	0	
Not identified		0.77	2.14	0.94	0.3	1.17	

Significant values are in [bold].

^a^Retention time.

^b^Relative content.

^c^Retention indices determined relative to n-alkanes (C7–C20) on a DB-5 GC Column.

**Table 3:** Chemical compounds of *Heracleum persicum* (HP), *Pimpinella anisum* (PA), *Kelussia odoratissima* (KO) and *Foeniculum vulgare* (FV) fruit essential oil.

Compounds name	RT (min)^a^	HP (%)^b^	PA (%)^b^	KO (%)^b^	FV (%)^b^	RI^c^
α-Pinene	8.69	–	–	–	1.79	938
Camphene	9.29	–	–	–	0.2	954
β-Pinene	10.27	–	–	0.33	0.34	980
Myrcene	10.48	–	–	–	0.87	984
Isobutyl-2-methyl butyrate	11.01	0.24	–	–	–	1004
α-Phellandrene	11.23	–	0.37	0.31	0.18	1010
Hexyl acetate	11.38	0.29	–	–		1014
α-Terpinene	11.66	–	–	–	0.13	1020
p-Cymene	12.18	–	–	0.36	0.15	1029
Limonene	12.14	–	**10.13**	**8.74**	**7.65**	1034
2-Methyl butyl isobutyrate	12.23	0.28	–	–	–	1037
1,8-Cineole	12.27	–	–	–	1.51	1038
Z-β-Ocimene	12.33	–	–	–	0.43	1040
Butyl-2-methyl butanoate	12.41	0.47	–	–		1042
γ-Terpinene	13.31	–	–	–	0.29	1063
3Z-Octen-1–01	13.35	0.39	–	–	–	1064
n-Octanol	13.76	**8.38**	–	–	–	1074
Fenchone	14.79		–	–	**16.75**	1092
2-Methyl butyl-2-methyl-butyrate	15.01	1.4	–	–	–	1101
Hexyl isobutanoate	16.89	1.45	–	–	–	1138
Camphor	17.29	–	–	–	0.3	1145
Pentyl cyclohexa-1,3-diene	17.56	–	–	1.72	–	1155
3Z-Hexanyl butanoate	19.12	2.65	–	–	–	1177
Methyl chavicol	19.38	–	2.27	–	**3.33**	1202
*trans*-Dihydro carvone	19.71	–	0.98	–	–	1221
1-Octyl acetate	19.9	**63.44**	0	–	–	1214
Hexyl-2-methyl butanoate	20.81	4.21	0	–	–	1236
Carvone	21.46	–	**9.15**	–	1.82	1291
p-Anis aldehyde	22.05	–	0	–	0.42	1260
Lavandulyl acetate	22.85	–	0	0.5	–	1286
(*E*)-Anethole	23.43	–	**75.43**	–	**63.35**	1290
Carvacrol	23.66	0.27	–	–	–	1300
Octyl isobutyrate	25.42	1.86	–	–	–	1334
α-Ylangene	26.73	–	–	0.62	–	1370
α-Copaene	27.02	–	–	2.63	–	1375
α-Cubebene	25.72	–	–	1.05	–	1385
β-Elemene	27.55	–	–	2.96	–	1390
Decyl acetate	28.15	1.09	–	–	–	1406
(E)-Caryophyllene	28.45	–	–	1.32	–	1415
Decenyl acetate	28.76	1.21	–	–	–	1422
Lavandulyl isobutanoate	28.79	–	–	0.8	–	1423
Octyl-2- methyl butyrate	29.15	6.03	–	–	–	1004
γ-Elemene	29.21	–	–	**5.07**	–	1433
α-Guaiene	29.44	–	–	0.45	–	1439
α-Humulene	30.2	–	–	0.66	–	1458
α-Acoradiene	30.34	–	–	0.77	–	1461
γ-Muurolene	31.13	–	–	**4.21**	–	1475
γ-Himachalene	31.27	–	1.15	–	–	1483
Germacrene D	31.49	–	–	**15.28**	–	1490
α-Zingiberene	31.73	–	0.52	–	–	1494
β-Selinene	31.75	–	–	2.2	–	1496
cis-β-Guaiene	32	–	–	2.87	–	1500
α-Muurolene	32.19	–	–	1.08	–	1505
Cuparene	32.31	–	–	0.7	–	1508
δ-Amorphene	32.45	–	–	0.54	–	1511
γ-Cadinene	32.63	–	–	1.81	–	1515
δ-Cadinene	32.84	–	–	5.9	–	1520
Selinene	33.3	–	–	**5.2**	–	1525
α-Cadinene	33.58	–	–	1.32	–	1536
Selina-3,7 (11)-diene	33.74	–	–	**3.14**	–	1540
Germacrene B	34.54	–	–	**7.45**	–	1557
Octyl hexanoate	35	1.22	–	–	–	1567
Dodecyl acetate	35.96	0.31	–	–	–	1603
3Z-Butyliden phthalide	38.69	–	–	2.19	–	1678
Z-Ligustilide	41.05	–	–	**15.18**	–	1738
Aliphatic ester		86.5	–	–	–	
Alcohol		8.38	–	–	–	
Monoterpene alcohol		0.27	–	–	–	
Aldehyde		–	–	–	0.42	
Alkene		–	–	1.72	–	
monoterpene ester		–	–	1.3	–	
Monoterpene hydrocarbons		–	10.5	9.74	12.03	
Oxygenated monoterpenoids		–	10.13	–	20.38	
Phenylpropanoids		–	77.7	–	66.68	
Phthalides		–	–	17.37	–	
Sesquiterpene hydrocarbons		–	1.67	67.23	–	
Not identified		4.85	–	2.64	0.49	

Significant values are in [bold].

^a^Retention time.

^b^Relative content.

^c^Retention indices determined relative to n-alkanes (C7–C20) on a DB-5 GC Column.

The main compounds of *A. graveolens* essential oil included limonene (monoterpene hydrocarbons), carvone (oxygenated monoterpenoids) and dill apiole (phenylpropanoids) in amounts of 33.12, 54.2 and 4.04%, respectively. α-pinene and β-pinene were the main terpene components identified in the essential oils of the two plants, and they constituted 22.83% and 48.36% of the total essential oil in *F. gummosa*, and 45.3% and 29.70% of the total essential oil in *P. crispum*, respectively (Table 2). δ-3-carene was also a member of the monoterpene hydrocarbon family and accounted for 10.69% of *F. gummosa* essential oil (Table 2). Phenylpropanoids constituted 11.22% of the essential oil of *P. crispum*. Indeed, myristicin was the most important phenylpropanoid in *P. crispum* (9.34%) (Table 2). In addition to high amounts of monoterpene hydrocarbons and oxygenated monoterpenoids, *C. cyminum* and *B. persicum* also had considerable amounts of aldehyde*.* The highest aldehyde content in these essential oils belonged to cuminaldehyde and γ-terpinen-7-al, which were about 13.92% and 15.34% in *C. cyminum* and 41.84% and 3.32% in *B. persicum*. Also, their main original monoterpene hydrocarbons were *p*-cymene, β-pinene, and γ-terpinene (Table 2). In comparison to *C. cyminum* (0.914 mg/µL), the density of *B. persicum* (0.876 mg/µL) essential oil was smaller (Table 2). sesquiterpene hydrocarbon is another fundamental family of the chemical profile of essential oils. They comprise about 67.2% *K. odoratissima* essential oil. The major chemical compounds of *K. odoratissima* essential oil encompassed δ-cadinene, selinene, germacrene D, and γ-elemene. limonene (monoterpene hydrocarbon), and Z-ligustilide (phthalides) were the two other major components constituting 8.74% and 15.18% of the essential oil of this plant, respectively (Table 3). *trans*-anethole is a phenylpropanoid whose amounts in the fruits of *P. anisum* and *F. vulgare* were 75.43 and 63.35% of essential oil, respectively (Table 3). Also, the essential oil of these plants had the highest mass-to-volume ratio (mg/μL) compared to other plants (Table 4). Other important compounds of these plants comprised limonene (10.13%), carvone (9.15%) in *P. anisum*, and fenchone (16.75%) in *F. vulgare* essential oils (Table 3). The classes of the compositions of *H. persicum* essential oil were totally different. In addition, this essential oil has the lowest density (0.766 mg/µL) among other studied species of Apiaceae. Most compounds of *H. persicum* essential oil belonged to aliphatic ester family. 1-octyl acetate, n-octanol, and octyl-2-methylbutyrate were the main components of *H. persicum* essential oil, which made up 63.44, 8.38, and 6.03% of the essential oil of the fruit of this plant, respectively (Table 3). In general, the main compounds and the range of essential oils amounts current study are in line with previous evaluating of *F. gummosa*^38^, *P. crispum*^39,40^, *A. graveolens*^41,42^, *C. Cyminum*^18^, *B. persicum*^43^, *H. persicum*^44^*, P. anisum*^45^, *K. odoratissima*^4^ and *F. vulgare*^24^ composition.

**Table 4:** Density and antioxidant activity of *Ferula gummosa* (FG)*, Petroselinum crispum* (PC)*, Anethum graveolens* (AG)*, Cuminum Cyminum* (CC)*, Bunium persicum* (BP), *Heracleum persicum* (HP)*, Pimpinella anisum* (PA)*, Kelussia odoratissima* (KO) and *Foeniculum vulgare* (FV) fruit essential oil.

	FG	PC	AG	CC	BP	HP	PA	KO	FV
Density (mg/µL)	0.859 ± 0.07	0.826 ± 0.01	0.873 ± 0.02	0.914 ± 0.02	0.876 ± 0.05	0.766 ± 0.01	0.932 ± 0.03	0.925 ± 0.01	0.990 ± 0.0
Reducing power EC_50_ (mg/mL)	83.38 ± 0.47	183.33 ± 0.54	> 200	161.19 ± 0.07	103.5 ± 0.62	> 200	> 200	43.22 ± 0.6	> 200
DPPH IC_50_ (mg/mL)	40.7 ± 0.23	22.13 ± 0.07	29.03 ± 0.11	15.17 ± 0.02	10.81 ± 0.58	45.94 ± 0.05	19.56 ± 0.02	78.14 ± 0.17	> 200

Mean ± standard error (n = 3).

### Antioxidant activity of fruits essential oil

We presented essential oil antioxidant activity through reducing power and DPPH methods. Through DPPH methods (IC_50_), the antioxidant potential of *F. gummosa* and *P. crispum* essential oils was evaluated at about 40.7 and 22.13 mg/mL (Table 4). Also, the reducing powers (EC_50_) of *F. gummosa* and *P. crispum* essential oils were estimated at about 83.38 and 183.33 mg/mL. Indeed, *F. gummosa* had a weaker DPPH radical scavenging capacity and stronger reducing power than *P. crispum*, while both of them contained high amounts of β-pinene and α-pinene (Table 4). Usually, the biological activity of essential oils varies in different plant species. At the same time, the difference in enantiomers content of a compound with different biological activity, such as α-Pinene and β-pinene enantiomers, have distinct properties^46^. The lowest antioxidant activity of essential oils belonged to *F. vulgare*. Also, *H. persicum* essential oil had a weaker antioxidant power than the most studied plants. The lowest consumption of essential oils for inhibiting 50% of DPPH (IC_50_) is related to *B. persicum* (Table 4). Therefore, in terms of scavenging DPPH radicals, the *B. persicum* essential oil (IC_50_: 10.81 mg/mL) has the highest potential. Afterwards*, C. cyminum* and *P. anisum* had a stronger antioxidant activity than others based on the DPPH method. The ranges of IC_50_, such as 114.87 to 287.56 µg/mL^45^ and 0.384 to 0.809 mg/mL^47^ were presented for the DPPH free radical scavenging capacity of the *P. anisum* essential oil. These findings are inconsistent with that of the present study. In a report released by Das et al.^48^, the IC_50_ of *P. anisum* (Varanasi, India) essential oil was equal to 12.31 µL/mL (for DPPH free radical scavenging), which is consistent with the finding obtained in this study (IC_50_ = 19.56 mg/mL), due to the range of density of the *P. anisum* essential oil (0.932 g/mL or mg/µL). The slight difference between them was due to differences in the content of constituents of essential oils. In previous studies, IC_50_ was reported to range from 7.9 to 13.6 mg/mL for DPPH scavenging capacity of *C. cyminum* essential oil^49^ and is in the acceptable range of the current study. It has been reported that among cumin essential oil compounds, the DPPH radical inhibitory power of γ-terpinene (IC_50_ = 22.73 mg/mL) was higher than those of other compounds such as cuminaldehyde (IC_50_ = 105.36 mg/mL), β-pinene (IC50 = 135.55 mg/mL) and *p*-cymene (IC_50_ = 122.25 mg/mL), and the scavenging power of the *C. cyminum* essential oil based on IC_50_ was 35.94 mg/mL^50^. Therefore, the antioxidant properties of essential oils such as *C. cyminum* and *B. persicum* can differ from the main pure compounds due to the interaction between the components and the chemical structure of the essential oil profile. Based on the DPPH method, the antioxidant power of *K. odoratissima* essential oil was weaker than those of all species (except *F. vulgare*). However, this plant has the highest reducing power with EC_50_ of 43.22 mg/Ml, while both of these methods are based on measuring the electron-donating capacity of an antioxidant. Therefore, there should not be huge contradictions in determining the antioxidant potential of several chemical compounds (like essential oil) in these methods. In some cases, the results of the reducing power method have little in consonance with the DPPH method regarding the antioxidant properties of these plants essential oils. For instance, according to the DPPH results, the essential oils of *C. cyminum*, *B. persicum*, and *P. anisum* show a high antioxidant power. However, the results obtained from their reducing power indicate a lower antioxidant power than some plants like *F. gummosa* and *K. odoratissima* (Table 4). In the reducing power methods, all reaction solutions were water-soluble. Owing to the nature of essential oils as nonpolar and hydrophobic molecules, the essential oil concentration was prepared in a non-aqueous solvent like methanol. When the reaction solution and different essential oil concentrations were mixed, an emulsion was formed to evaluate the reducing power of essential oils. It has been proven that different types of chemical compositions of essential oils affect the quality and stability of emulsions formed^51,52^. Therefore, unlike the DPPH method, the reducing power of the essential oil, in addition to its antioxidant power, is influenced by the quality and stability of the emulsion in different plants. This approach makes some misinterpretations in the antioxidant power of essential oils in this method. For this reason, unlike the antioxidant report of the methanol extract of the fruit (Table 1), the antioxidant power results of the essential oil via DPPH and power reduction methods could not be matched. Furthermore, the evaluation of the reducing power of essential oils at concentrations above 200 mg/mL was inestimable due to the instability of the emulsions created in the reaction solution. On the other hand, in the essential oils of *A. graveolens*, *P. anisum*, *F. vulgare*, and *H. persicum*, the increasing trend of reducing power was insignificant with an increasing concentration; therefore, EC_50_ (absorption 0.5) was not calculated (Table 4). In the previous study, it has been reported that in a comparison between the essential oils of 6 medicinal plants and their 5 main compounds, exactly the same trends (i.e., stability in absorption along with increasing concentration) were observed in *trans*-anethole and *F. vulgare*. Thus, the antioxidant activity did not increase with the increase in the concentration of anethole and *F. vulgare* essential oil^53^.

### Antimicrobial activity

In the current study, positive controls were Gentamicin and Ciprofloxacin for antibacterial properties and Voriconazole for antifungal properties. As shown in Table 5, *P. aeruginosa* is the most resistant bacterium to the used chemical antibiotics. Furthermore, *P. aeruginosa* has more resistance to essential oils than other bacteria. *P. aeruginosa* species has a high resistance to external factors due to having a lipopolysaccharide and phospholipid membrane^54^. Also, the lipopolysaccharide membrane plays an essential role in the resistance of this bacterium to hydrophobic antibacterial compounds (e.g., essential oils)^55^.

**Table 5:** Antimicrobial activity chemical antibiotics.

Antibiotic agents		Bacteria				Fungi	
		Gram-positive		Gram-negative		Yeast	Mold
		*B. subtilis*	*S. aureus*	*E. coli*	*P. aeruginosa*	*C. albicans*	*A. flavus*
Cip	IZ (mm)	22.93 ± 0.33	23.63 ± 0.22	23.66 ± 0.56	20.94 ± 0.11	NT	NT
	MIC (mg/mL)	0.0075	0.0075	0.0075	0.01	NT	NT
	MBC (mg/mL)	0.01	0.01	0.01	0.02	NT	NT
Gen	IZ (mm)	17.34 ± 0.24	15.5 ± 0.11	25.25 ± 0.21	13.51 ± 0.67	NT	NT
	MIC (mg/mL)	0.03	0.01	0.007	0.03	NT	NT
	MBC (mg/mL)	0.03	0.015	0.007	0.06	NT	NT
Vori	IZ (mm)	NT	NT	NT	NT	28.12 ± 0.09	21.62 ± 0.83
	MIC (µg/mL)	NT	NT	NT	NT	0.125	0.125
	MFC (µg/mL)	NT	NT	NT	NT	1.25	1.5

Diameter of inhibition zone including disc diameter of 6 mm. Mean ± standard error (n = 3).

*NT* Not tested, *IZ* Inhibition zone, *Gen* Gentamicin (10 µg per disk), *Cip* Ciprofloxacin (5 µg per disk), *Vori* Voriconazole (1 µg per disk), *MIC* Minimum inhibitory concentration, *MBC* Minimal bactericidal concentration, *MFC* Minimal fungicidal concentration.

In general, there are no detailed reports on the antimicrobial properties of some wild plants, such as *F. gummosa*, *H. persicum* and *K. odoratissima*. However, in Table 6, a brief report of the range of antimicrobial properties of the essential oils of Apiaceae family plants was obtained based on MIC and MBC. What is evident in the presented table is that the range of estimated values is vastly contradictory. In fact, based on a general summary of previous research results, it is impossible to distinguish the antimicrobial power of each plant's essential oil, either separately or comparatively within the family. In previous studies, the range of MIC and MBC values exists as values with units of µg/mL and mg/mL in some species (Table 6). Although the results can be different due to the difference in the bacterial strains and the chemical profile of the plants, many of the observed differences are beyond these factors. Due to the liquid nature of essential oils, it is common to report MIC and MBC values as µL/mL. However, special attention should be paid to the density of essential oils to express MIC and MBC based on µg/mL and mg/mL. As presented in Table 4, each essential oil has a different density according to the its constituent compounds. For example, according to Table 7, the MIC value in the essential oil of *H. Persicum* and *F. vulgare* plants against *A. flavus* was estimated as 1 mg/ML. According to the density of the essential oil of *H. Persicum* (0.766 mg/µL) and *F. vulgare* (0.990 mg/µL), this value is equivalent to 1.30 and 1.01 µL/mL, respectively. The lack of attention to the density and the approach expressed regarding unit conversion are the main reasons for the difference in various reports. In addition, a numerical range regarding the antimicrobial properties of the main purified compounds related to the essential oils of this family, such as α-pinene, limonene, carvone, *trans*-anethole and ligustilide has also been presented in Table 6. This can provide an overview of the range of antimicrobial effects of the main essential oil compounds of this family in the form of MIC and MBC values. In this study, according to the results of a wide range of effective concentrations in previous studies, the range of concentrations from 0.005 mg/mL (equivalent to 5 µg/mL) to 96 mg/mL was tested to enable the conduct of accurate evaluation and measurements. According to Tables 7 and 8*A. graveolens*, *K. odoratissima* and *H. persicum* have acceptable antibacterial properties. Regarding the results of MIC, MBC, and inhibition zone, the antibacterial effect of *K. odoratissima*, particularly against *B. subtilis* and *E. coli* is substantial. Nevertheless, despite the positive antibacterial response of *H. persicum* and *A. graveolens* essential oil, their antibacterial properties compared to other studied plants have intermediate positions. Against similar studied bacteria, Ruangamnart et al.^26^ reported that the range of MIC of *A. graveolens* essential oil (containing 44.61% limonene, 28.02% carvone and 19.98% dillapiole) was higher than 10 mg/Ml. While the reported chemical profile was partly different from that of the current study, especially in terms of dillapiole content. limonene constitutes 33.12% of the essential oil of *A. graveolens* and 8.74% of the essential oil of *K. odoratissima*. However, the antibacterial properties of this compound through the MIC method were reported to be approximately 16 µL/mL against *E. coli*^72^. In another study, the reported MIC values of 26.25 and 52.5 mg/mL for *E. coli* and *S. aureus* were claimed, respectively^73^. Carvone was another main *A. graveolens* essential oil compound (54.3%) and its MIC against *S. aureus* (ATCC 6538) and *B. subtilis* (ATCC 6633) with the same strains of the current study were evaluated to be about 5 and 10 mg/mL^22^.

**Table 6:** Summary of the MIC and MBC range of some Apiaceae essential oils and their main compounds against some pathogenic microorganisms in previous studies.

Plant/compounds references	Microorganism	Biological properties
*B. persicum*^56^	*Streptococcus pyogenes, Staphylococcus epidermidis, Escherichia coli, Shigella flexneri, Pseudomonas aeruginosa* and *Enterobacter aerogenes*	MIC: 1–8 mg/mL MBC: 1–16 mg/mL
	*Candida albicans*	MIC: 1 mg/mL MFC: 1 mg/mL
*B. persicom*^57^	*Pseudomonas tolaasii*	MIC: 3.12 µg/mL MBC: 1.25 µg/mL
*F. vulgare*^58^	*Staphylococcus epidermidis, Staphylococcus aureus, Pseudomonas aeruginosa* and *Proteus mirabilis*	MIC: 250 to > 2000 µg/mL
*F. vulgare*^25^	*Staphylococcus aureus* and *Pseudomonas aeruginosa*	MIC: In both > 10 mg/mL MBC: NP
*F. vulgare*^58^	*Escherichia coli* and *Bacillus subtilis*	MIC: 295.5 and 62 mg/mL MBC: NP
*C. cyminum*^22^	*Staphylococcus aureus, Listeria monocytogenes, Escherichia coli* and *Salmonella enteritidis*	MIC: 0.75–3 mg/mL MBC: 1.5–3 mg/mL
*C. cyminum*^23^	*Staphylococcus aureus, Bacillus cereus, Listeria monocytogenes, Escherichia coli* and *Salmonella typhimurium colony*	MIC: 12.5–50 µg/mL MBC: 25–100 µg/mL
*A. graveolens*^26^	*Staphylococcus aureus, Streptococcus pyogenes, Escherichia coli* and *Pseudomonas aeruginosa*	MIC: In all > 10 mg ml MBC: In all > 10 mg ml
*A. graveolens*^27^	*Bacillus cereus, Staphylococcus aureus, Pseudomonas aeruginosa* and *Escherichia coli*	MIC: 5–40 µg/mL MBC: 10–80 µg/mL
	*Aspergillus fumigatus* and *Candida albicans*	MIC: 20 and 10 µg/mL MFC: 40 and 20 µg/mL
*P. anisum*^59^	*Staphylococcus aureus, Bacillus cereus, Escherichia coli* and *Pseudomonas aeruginosa*	MIC: 62.5 to > 500 µg/mL MBC: NP
*P. anisum*^60^	*Staphylococcus aureus, Streptococcus pyogenes, Escherichia coli* and *Pseudomonas aeruginosa*	MIC: 2.5–4 µg/mL MBC: NP
	*Candida albicans*	MIC: 4 µg/mL MFC: NP
*P. anisum*^61^	*Clostridium perfringens*	MIC: 10 mg/mL MBC: 20 mg/mL
*P. anisum*^62^	*Staphylococcus aureus* and *Escherichia coli*	MIC: 3.1 and 4 µL/mL
*P. anisum*^63^	*Escherichia coli, Listeria monocytogenes, Staphylococcus aureus* and *Pseudomonas fluorescens*	MIC: 1.25 and 2.5%V/V MBC: > 5%V/V
(+)-α-Pinene^64^	*Staphylococcus aureus* and *Escherichia coli*	MIC: 2.5 and 1.25 µL/mL MBC: 10 and 1.25 µL/mL
(−)-α-Pinene^65^	57 *Campylobacter jejuni strains*	MIC (in all cases): 1000, 2000 and > 2000 mg/mL
α-Pinene^66^	*Staphylococcus aureus* and *Escherichia coli*	MIC: 0.420 and 0.686 mg/mL MBC: 1.716 and 3.432 mg/mL
Limonene^66^	*Staphylococcus aureus* and *Escherichia coli*	MIC: 0.421 and 0.421 mg/mL MBC: 1.682 and 1.682 mg/mL
Carvone^67^	*Staphylococcus aureus*	MIC: 2 mg/mL
*trans*-Anethole^68^	*Staphylococcus aureus*	MIC: 4 < %
δ-3-Carene^69^	*Staphylococcus aureus, Bacillus cereus,* and *Escherichia coli*	MIC: 0.16 mg/mL
Ligustilide^70^	*Staphylococcus aureus* and *Bacillus cereus*	MIC: In both 1 mg/mL
(−)-carvone^71^	*Staphylococcus aureus, Bacillus cereus, Escherichia coli* and *Pseudomonas aeruginosa*	MIC: 2.5–5 mg/mL MBC: 5 to > 20 mg/mL
	*Candida albicans*	MIC: 2.5 mg/mL MFC: 10 mg/mL

*NP* not presented.

The current result also demonstrates that the inhibitory and bactericidal power of *A. graveolens* essential oil against *S. aureus* is better than *B. subtilis*; and the range of MIC and MBC has consonance with the biological activity of its main compounds. In the case of limonene, in addition to the antibacterial effects of the compound itself, high synergistic effects were observed between the compound and chemical antibiotic compounds against several bacterial species such as *E. coli* and *S. aureus*^74^. The presence of limonene, along with other main components of *K. odoratissima* essential oil, may increase the synergetic effects and consequently increase the antibacterial properties. As it was mentioned above, there are no specific reports about the antibacterial properties of *K. odoratissima* and *H. persicum*; however, the biological properties of any essential oil are directly related to its components. For instance, *K. odoratissima* contains a high amount of Z-ligustilide and its MICs were evaluated to be 1, 1, 8 and 10 mg/mL against *B. subtilis*, *S. aureus*, *E. coli* and *P. aeruginosa*, respectively (Table 8). Previously, it has been reported that Z-ligustilide compound purified from *Cnidium officinale* essential oil had high antibacterial properties, and its MIC against *S. aureus* was 1 mg/mL^70^. The amount of MIC in *H. persicum* essential oil was estimated to be 8, 10, 8, and 24 mg/mL against *B. subtilis*, *S. aureus*, *E. coli* and *P. aeruginosa*, respectively (Table 8). Its essential oil consists of high amount of acetate composition and octanol. These compounds such as octyl acetate and octanol were factors of antibacterial properties in the *Zosima absinthifolia* plant^75^. In the past, the concentrations of MIC of *Heracleum rigens* essential oil (containing 51.2% bornyl acetate, 9.62% limonene, and 3.94% octyl acetate) for *S. aureus*, *Bacillus subtillis*, *E. coli*, and *P. aeruginosa* were estimated to be 4.5, 2.25, 36, and 36 mg/mL, respectively. Based on MIC and MBC results, *P. crispum*, *P. anisum*, and *F. vulgare* essential oil up to 96 mg/mL had no antibacterial power on the tested bacteria (Tables 8). Furthermore, these essential oils inhibited the lowest zone (Table 7). Even the essential oil of *P. crispum* and *P. anisum* against *B. subtilis* bacterium did not form any inhibition zone. Approximately 75 and 63% of the essential oil of *P. anisum* and *F. vulgare* were *trans*-anethole. In the past, it has been determined that *trans*-anethole isolated from the *Croton zehntneri* plant did not have antibacterial properties against *S. aureus*, *P. aeruginosa*, and *E. coli* bacteria^76^. Also, the previous evaluation of *trans*-anethole activity against *S. aureus* indicated that this compound had a weak antimicrobial potential^68^. Presumably, *trans*-anethole has a non-toxic effect on bacteria so it can be the reason for the lack of antibacterial responses in the *P. anisum* and *F. vulgare* essential oil. Similar to the results of the current study, Fitsiou et al.^77^ studied the essential oils of several medicinal plants and found that the *P. anisum* essential oil did not have any antibacterial activity. On the contrary, there were reports of antibacterial properties of the essential oil of this plant on the same bacteria, MIC in the range of 0.06 to 0.25 µg/mL^78^ and 2.0 to 3.0 µg/mL^60^ were reported, which were inconsistent with the results of this study.

**Table 7:** Antimicrobial activity (inhibition zone) of *Ferula gummosa* (FG)*, Petroselinum crispum* (PC)*, Anethum graveolens* (AG)*, Cuminum Cyminum* (CC)*, Bunium persicum* (BP), *Heracleum persicum* (HP)*, Pimpinella anisum* (PA)*, Kelussia odoratissima* (KO) and *Foeniculum vulgare* (FV) fruits essential oil.

Microorganisms	FG	PC	AG	CC	BP	HP	PA	KO	FV
Gram positive									
*B. subtilis*	17.78 ± 0.97	6.00	15.20 ± 0.25	25.53 ± 0.56	26.33 ± 0.56	11.60 ± 0.24	6.00	24.92 ± 0.49	10.62 ± 0.97
*S. aureus*	24.68 ± 0.54	10.27 ± 0.35	15.16 ± 0.18	24.88 ± 0.65	25.23 ± 0.65	12.18 ± 0.42	10.7 ± 0.12	13.80 ± 0.83	10.88 ± 0.29
Gram negative									
*E. coli*	23.39 ± 0.46	11.41 ± 0.43	16.75 ± 0.57	31.02 ± 0.84	25.60 ± 0.41	9.79 ± 0.29	10.56 ± 0.20	28.97 ± 1.18	13.22 ± 0.51
*P. aeruginosa*	20.10 ± 0.79	12.84 ± 0.59	19.83 ± 0.77	37.23 ± 1.25	39.33 ± 1.25	13.01 ± 0.37	13.14 ± 0.33	19.77 ± 0.94	9.78 ± 0.30
Fungus									
*C. albicans*	17.15 ± 0.73	27.54 ± 0.15	19.79 ± 0.74	41.28 ± 0.76	41.28 ± 0.76	28.62 ± 0.96	12.87 ± 0.67	15.56 ± 0.51	12.84 ± 0.47
*A. flavus*	9.22 ± 0.84	9.24 ± 0.92	10.28	14.12	12.22	16.45 ± 0.79	17.34 ± 1.12	12.14 ± 0.54	16.2

Diameter of inhibition zone including disc diameter of 6 mm. Mean ± standard error (n = 3).

*NT* Not tested, *Gen* Gentamycin (10 µg per disk), *Cip* Ciprofloxacin (5 µg per disk), *Vori* Voriconazole (1 µg per disk).

According to the results of Table 2, the predominant compounds of *F. gummosa* and *P. crispum* essential oils are two common compounds of α-pinene and β-pinene. However, there are significant differences between antibacterial properties of these essential oils in terms of MIC, MBC and inhibition zone traits. The weak antibacterial properties of *P. crispum* essential oil have previously been revealed^79^. In contrast, the amount of inhibition zone of *F. gummosa* essential oils indicated strong antibacterial properties. Similarly, these estimations indicate its MIC and MBC value against *B. subtilis*, *S. aureus*, and *E. coli* was 2 mg/mL. The possibility of differences in the enantiomeric profile of the main components of the essential oils of these plants is a major reason for this difference. It was reported that differences in levels of enantiomeric compositions of α-pinene ((1R)-(+)-α-pinene and (1S)-(−)-α-Pinene) in *Juniperus communis* essential oil could increase its lethality effects by 4 to 16 times. It has also been reported that (−)-α-pinene, as an enantiomeric composition of α-Pinene, has had no antibacterial or antifungal properties; however, (+)-α-pinene has had strong antimicrobial properties^80^. Therefore, the amounts of pinene detected in the essential oils of the two different plants, including *F. gummosa* and *P. crispum*, might have different chemical reactions due to the differences in the enantiomeric compositions of Pinene. Also, the most enantiomeric composition of α-pinene in *F. gummosa* essential oil has been reported to be (1R)-(+)-α-pinene^38^.

**Table 8:** Antimicrobial activity (MIC and MBC) of *Ferula gummosa* (FG)*, Petroselinum crispum* (PC)*, Anethum graveolens* (AG)*, Cuminum Cyminum* (CC)*, Bunium persicum* (BP), *Heracleum persicum* (HP)*, Pimpinella anisum* (PA)*, Kelussia odoratissima* (KO) and *Foeniculum vulgare* (FV) fruits essential oil.

Microorganisms	FG (mg/mL)		PC (mg/mL)		AG (mg/mL)		CC (mg/mL)		BP (mg/mL)		HP (mg/mL)		PA (mg/mL)		KO (mg/mL)		FV (mg/mL)	
	MIC	MBC	MIC	MBC	MIC	MBC	MIC	MBC	MIC	MBC	MIC	MBC	MIC	MBC	MIC	MBC	MIC	MBC
Gram positive																		
*B. subtilis*	2	2	> 96	> 96	10	24	0.5	1	0.5	1	8	12	> 96	> 96	1	1	> 96	> 96
*S. aureus*	2	2	> 96	> 96	8	12	1	1	1	4	10	24	96	96	4	10	> 96	> 96
Gram negative																		
*E. coli*	2	2	> 96	> 96	12	48	1	1	2	4	8	12	> 96	> 96	1	2	> 96	> 96
*P. aeruginosa*	8	24	> 96	> 96	8	24	4	10	4	6	24	96	> 96	> 96	10	24	> 96	> 96
Fungus	MIC	MFC	MIC	MFC	MIC	MFC	MIC	MFC	MIC	MFC	MIC	MFC	MIC	MFC	MIC	MFC	MIC	MFC
*C. albicans*	0.5	2	4	6	96	> 96	0.5	0.5	0.25	0.25	0.25	0.5	48	48	4	10	24	48
*A. flavus*	4	96	4	24	4	4	2	4	1	2	1	1	1	4	2	4	1	4

*NT* Not tested, *MIC* Minimum inhibitory concentration, *MBC* Minimal bactericidal concentration, *MFC* Minimal fungicidal concentration.

Interactions and combined effects of all the essential oil components could be another reason for the differences in the properties of *F. gummosa* and *P. crispum* essential oils. This is due to the fact that the final properties of each essential oil are affected by all essential oil compounds and their antagonist and synergetic effects. For example, it is reported that (−)-α-pinene alone does not have high antibacterial properties; however, its use in combination with chemical antibiotics increases their antibacterial power by several hundred times^65^. Therefore, the interaction of the chemical components of the essential oil leads to differences in their properties due to the different chemical profile of each species. The δ-3-Carene compound is an active compound against bacteria with a MIC of about 0.16 g/L (equivalent to 0.16 mg/mL) against *E. coli*, *B. cereus*, and *S. aureus*^69^. It has also been determined that the reason for the high antibacterial power of 3-Carene compound can be attributed to the multiple mechanisms of its destructive effects on the morphological, physiological and molecular levels of bacteria. At the morphological level, this compound causes changes in the natural shape and structure of bacteria, disruption and destruction of bacterial membrane integrity, and leakage of intracellular substances. At the physiological levels, it reduces the metabolic function by impairing the ATP breakdown, intracellular Tricarboxylic acid cycle, and Glycolysis-related enzymes. At the molecular level, this compound binds to DNA, disrupts gene expression, and as a result, increases the antibacterial effect^81^. Therefore, the presence of this compound in *F. gummosa* essential oil, along with other essential oil components, can be a significant reason for the higher potency of its antibacterial properties than *P. crispum* essential oil (Tables 7, 8). The Antibacterial properties of *C. cyminum* and *B. persicum* were higher than others in all bacterial species based on the inhibition zone, MIC, and MBC. The concentration ranges of MIC, MBC, and inhibition zone for *C. cyminum* essential oil were 0.5–4, 1–10 mg/mL, and 24.88–37.23 mm and for *B. persicum* were 0.5–4, 1–6 mg/mL, and 25.23–39.33 mm, respectively (Tables 7, 8). In relation to the antibacterial properties of *C. cyminum* and *B. persicum* essential oils in the current study, the range of MIC and MBC had consonance with previous research findings. However, the expected difference could be attributed to differences in essential oils compounds and differences in the resistance of bacterial strains. Among the studied plants, *H. persicum*, *C. cyminum* and *B. persicum* essential oils had high antifungal activity against both *A. flavus* and *C. albicans*. In contrast, the essential oils of *A. graveolens*, *P. anisum* and *F. vulgare* had the lowest antifungal activity against *C. albicans* (Tables 7, 8). The essential oils of *F. gummosa* and *P. crispum* showed inhibition against *A. flavus* in 4 mg/mL concentration, but it seems that they had the lowest fungicidal properties against this fungus. Generally, it has been founded that since plant essential oils have hydrophobic components, they degrade and disrupt the membrane fluidity, change ergosterol biosynthesis, increase leakage, and change pH in the cytosolic space. Thus, they cause damage to cell organs and lead to the death of fungal and yeast cells^5^. In addition, the results showed that different compounds of the essential oils of each plant have different effects on different microorganisms. For example, it has been reported that α-pinene has no strong fungicidal effect against *A. flavus*^82^. According to the results of one study on the fungicidal effect of α-Pinene, the estimated amount of the MFC for *A. flavus* for this compound was approximately 15 times higher than the MFC for *C. albicans*^80^. Considering the amount of pinene in the essential oils of *P. crispum* and *F. gummosa*, it can be a reason for the superiority of these essential oils in the inhibition and lethality power of *C. albicans* compared to *A. flavus*. According to another report, cuminaldehyde has a high potency in damaging fungal hyphae, reducing ergosterol biosynthesis, growth inhibition, and aflatoxin production in *A. flavus*, as the MIC of fungal growth in this compound is about 0.64 μL/mL (equal to 0.62 mg/mL considering a density of about 0.97 g/mL for this compound)^83^. Previously, Das et al.^48^ have indicated the power of *P. anisum* essential oil in inhibiting *A. flavus*. anethole has been the main component of a *P. anisum* and *F. vulgare* essential oil. It has also been previously reported that the antifungal properties of *Illicium verum* and *F. vulgare* essential oils can be attributed to the high amount of anethole^84,85^.

**Figure 1:**
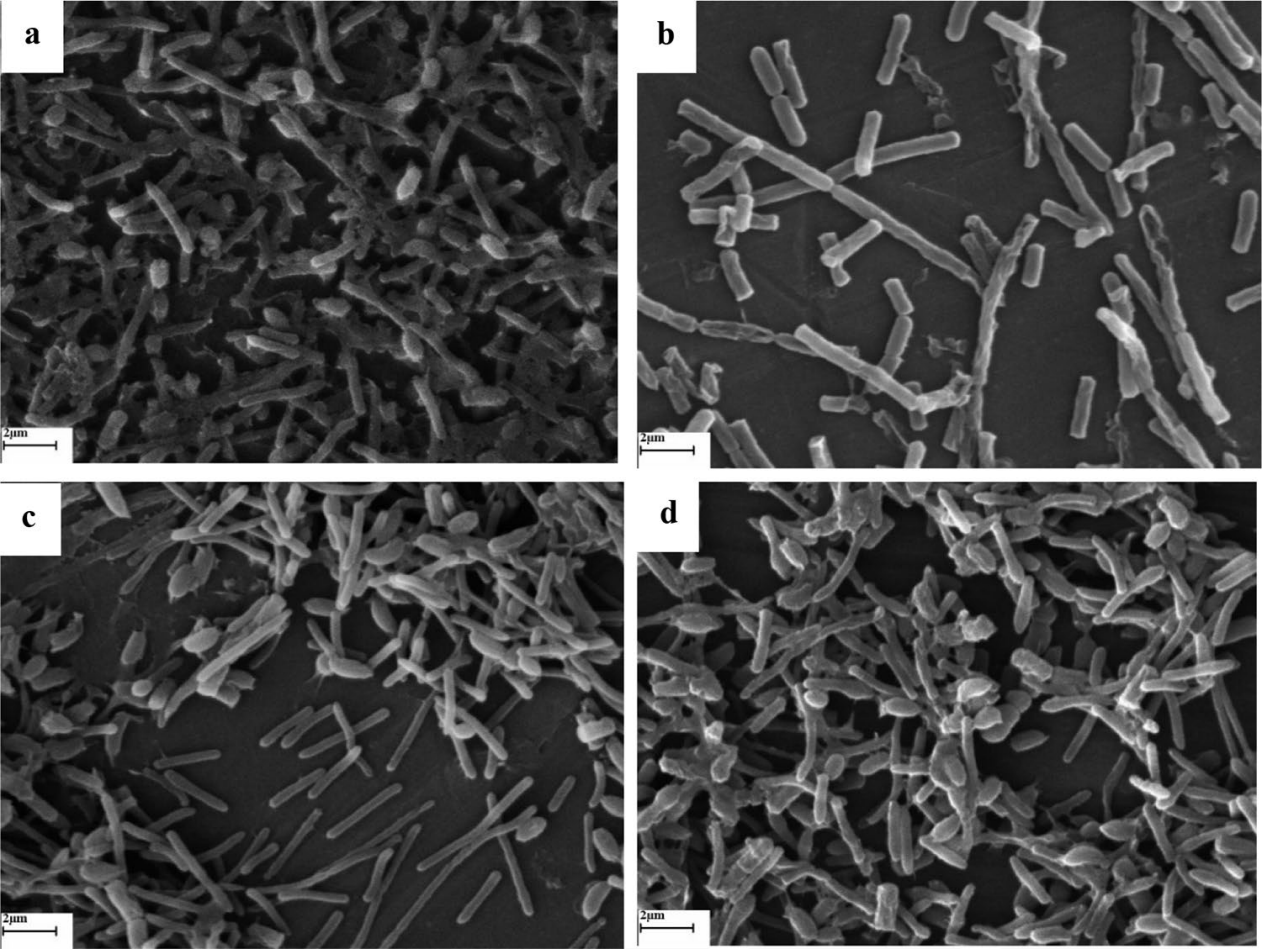
Scanning electron micrographs of the effect of *Cuminum cyminum* (**a**), *Heracleum persicum* (**b**), *Anethum graveolens* (**c**) and *Petroselinum crispum* (**d**) essential oils on *B. subtili*.

**Figure 2:**
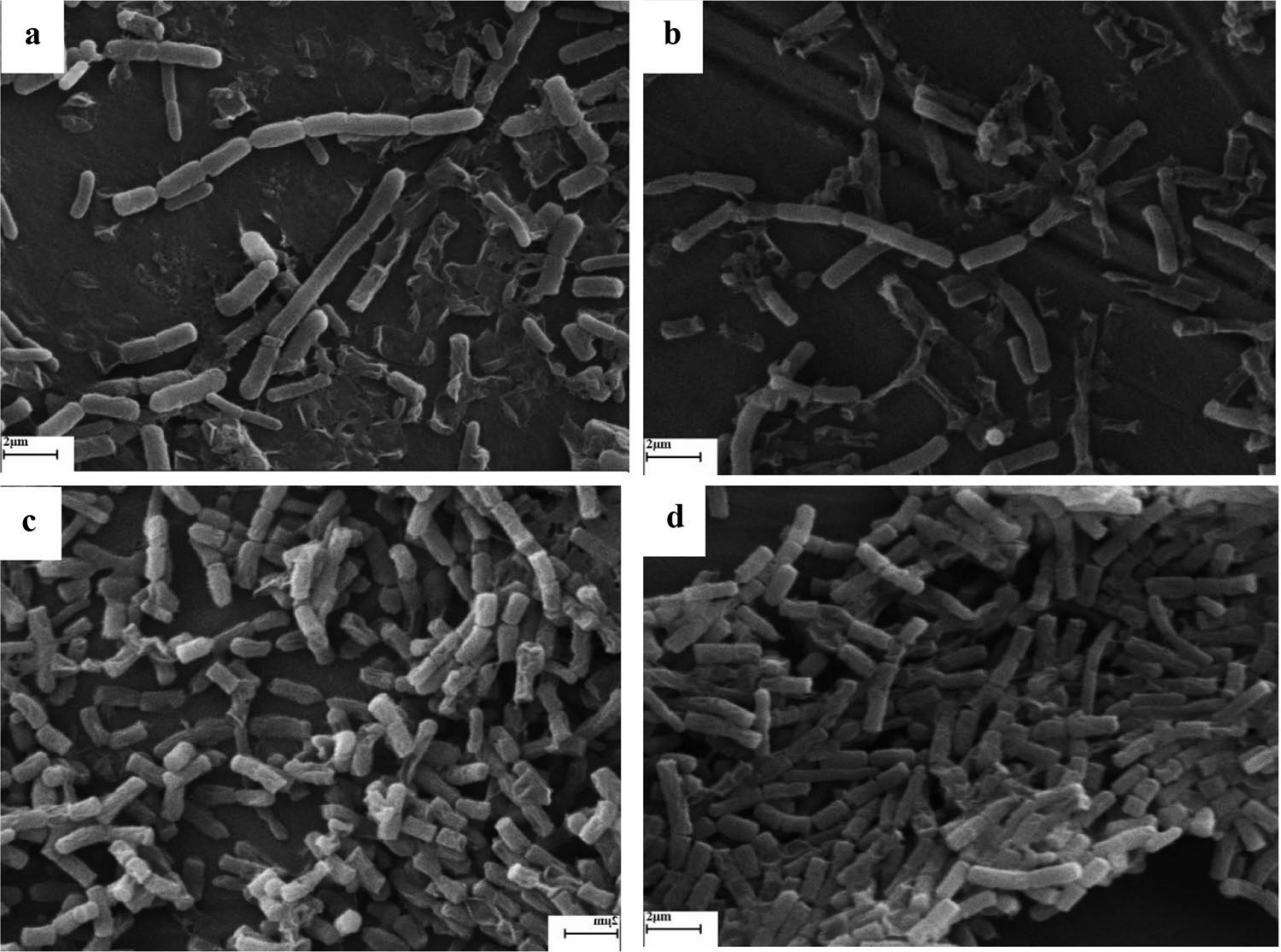
Scanning electron micrographs of the effect of *Cuminum Cyminum* (**a**), *Heracleum persicum* (**b**), *Anethum graveolens* (**c**) and *Petroselinum crispum* (**d**) essential oils on *E*. *coli*.

Related research findings have revealed that the membranes and walls of bacteria are destroyed by plant essential oils. However, other destructive effects such as cytoplasmic coagulation, cytoplasmic content leakage, and protein damage have also been reported against bacteria with essential oil application^86^. One of the reasons for the destruction of the bacterial membrane is the hydrophobic nature of essential oils, which disrupts the two phospholipid layers of the bacterial membrane, although the destructive effects of each essential oil on bacteria may be different depending on the type and composition of each essential oil^6^. All the SEM images show the effect of membrane degradation on both bacterial species with the application of *H. persicum* and *C. cyminum* essential oils (Figs. 1, 2). In SEM images, some membrane destruction can be detected in both species of bacteria with the use of *H. persicum* essential oil, but the SEM imaging of *C. cyminum* show that the power of *C. cyminum* essential oil in membrane destruction is much more intense. In addition, the effects of *C. cyminum* on the destruction of each of the two bacteria, especially *B. subtili*, can be regarded as a mass of damaged cell membranes. Some bacterial cells with disrupted membranes can be observed in *A. graveolens* treatments. But the effects of *A. graveolens* essential oil were lower than those of *H. persicum* and *C. cyminum*. In addition, no membrane degradation by *P. crispum* essential oil was not observed. Nevertheless, the essential oil of this plant caused morphological changes in bacteria, including changes in the size of the bacteria and shrinkage of the bacterial membrane, especially *E. coli*. (Figs. 1, 2).

### Principal component analysis (PCA) and agglomerative hierarchical clustering (AHC) on essential oils’ profiles and properties

**Figure 3:**
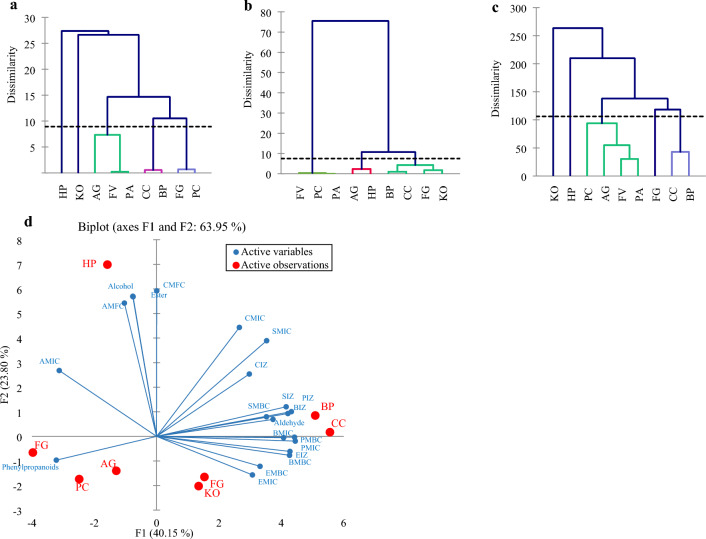
(**a**) Analysis based on features related to the chemical profile, (**b**) Analysis based on features related to the antimicrobial properties, (**c**) Analysis based on features related to the antimicrobial properties and chemical profile. (**d**) Principal Component Analysis based on antimicrobial activity of studied plants and their main compound groups. In figures, HP: *Heracleum persicum*, KO: *Kelussia odoratissima*, AG: *Anethum graveolens*, FV: *Foeniculum vulgare*, PA: *Pimpinella anisum*, CC: *Cuminum Cyminum*, BP: *Bunium persicum*, FG: *Ferula gummosa* and PC: *Petroselinum crispum*. BMIC, BMBC and BIZ: MIC, MBC and inhibition zone of *B. subtilis*, EMIC, EMBC and EIZ: MIC, MBC and inhibition zone of *E. coli*, BMIC, BMBC and BIZ: MIC, MBC and inhibition zone of *B. subtilis*, SMIC, SMBC and SIZ: MIC, MBC and inhibition zone of *S. aureus*, PMIC, PMBC and PIZ: MIC, MBC and inhibition zone of *P. aeruginosa*, CMIC, CMFC and CIZ: MIC, MFC and inhibition zone of *C. albicans* and AMIC, AMFC and AIZ: MIC, MFC and inhibition zone of *A. flavus*.

AHC analysis is a statistical approach to categorizing the tested factors based on their characteristics. In the current research, this analysis was done three times based on essential oil compounds data (Fig. 3a), antimicrobial properties data (Fig. 3b), and data related to essential oil compounds and antimicrobial properties together (Fig. 3c). As presented in Fig. 3a, the essential oils of KO and HP plants are more distinct from other plants in terms of chemical profile, and both of them form a cluster independently. FG and PC with a high level of pinene and BP and CC with a high level of Aldehyde compounds form the other two clusters. Finally, PA and FV with high similarity to each other and together with AG formed a cluster (Fig. 3a). In Fig. 3b, it has been shown that there are three classifications based on antimicrobial properties. The cluster has a group with high antimicrobial properties, including CC, BP, FG and KO; a group with moderate antimicrobial properties, including HP and AG; and a group with weak antimicrobial properties, including FV, PA and PC. Clearly, the best and most accurate classification was obtained based on the analysis of all data related to essential oil compounds and biological activities (Fig. 3c).

As a result, the differences between the plants have been determined more precisely in Fig. 3c based on essential oil compounds and biological activities and, thereby, a more complete and accurate view has been presented. The identification of compounds or common tests to evaluate the biological properties of essential oils is a costly, time-consuming, and sometimes dangerous process. On this basis, there is the possibility that a mathematical prediction model in the future can predict the properties of essential oils based on their constituent compounds. Since there are valuable differences and similarities in the grouping of Fig. 3a, b with Fig. 3c. for example, in all three modes of analysis, CC and BP have been placed in one group, and PA and FV have been placed in another group (Fig. 3a–c). This shows that it is possible to determine the potential of antimicrobial properties using modeling in advance, particularly in intra-familial classes based on the chemical composition of these plants. Reyes-Jurado et al.^87^ clearly state that common laboratory tests regarding the antimicrobial properties of essential oils are influenced by many variables such as human errors, source of essential oil, extraction method, amount of chemical compounds, the volume of inoculation, growth phase of microorganism, the strain of microorganisms to be tested, the culture medium used, the incubation time, the temperature, and the pH of water. Finally, according to the impact of the mentioned cases, there are distinctions and differences in the reports, even about a plant species (Table 6). As a result, as in the present study, one of the most effective methods to reduce these differences is to examine several plant species simultaneously and use techniques such as SEM to confirm the results of tests that rely solely on visual observations (such as MIC, MBC and inhibition zone). In this regard, the factors causing differences during the tests become uniform and, thereby, accurate comparisons are made with less error. However, the use of a mathematical model seems to be a more appropriate solution. Today, the mathematical modeling of chemical antibiotics is done in very advanced fields to check the effective dose even in the human body^88^. At the same time, there is no report regarding the application of these methods to investigate plant compounds' properties, even in vitro. In the present study, advanced modeling was not applicable due to the small number of tested samples. Therefore, the extent and number of laboratory samples in the future should be considered. Also, the placement of KO and HP in separate groupings in different states in AHC analysis shows that plant species should be selected based on similarities in chemical profiles to design the prediction model in the future. From each chemical profile with similar compositions, there should be several plant species for the conduct of a more accurate modeling. Accordingly, very contradictory chemical profiles should be removed from the prediction model. Another apparent contradiction is the position of FG in these groupings. Indeed, in Fig. 3a, the high level of pinene placed this plant in the same group as PC plant. While in their grouping based on antibacterial properties, the position of these two plants differed (Fig. 3a). In Fig. 3c, the FG plant was also placed in a group alone. It was pointed out that the enantiomers of a compound such as α-pinene have different biological properties. This trend shows that the difference in biological properties of enantiomeric composition can cause errors in the model.

Therefore, for the accomplishment of more accurate modeling, the enantiomeric composition levels of the main components of the essential oil should be well estimated. Based on the PCA analysis (Fig. 3d) and the location of independent variables (active observation), a high level of similarity between the essential oils of CC and BC plants and FG and PA plants was also observed. In the PCA diagram, the more acute the angle between the two factors, the stronger and more positive the correlation between the two factors. In this regard, the aldehyde compounds in the Apiaceae family can be considered potent bactericidal agents. Additionally, according to the distance of the traits indicating antibacterial properties (active variables) in different plants (active observation), the essential oils of two plants CC and BP (containing a high level of aldehyde compounds), had high antibacterial properties. Furthermore, the ester and alcohol compounds of plant essential oils are highly effective in killing fungi. HP plant essential oil shows the most fungicidal effect on these microorganisms.

## Conclusion

Overall, the results of measuring the methanol extract of fruits by DPPH and reducing power methods showed that the fruits of *F. gummosa*, *C. cyminum* and *P. anisum* plants had higher antioxidant properties than other studied plants. According to the DPPH method, the antioxidant properties of essential oil *C. cyminum* and *B. persicum* were stronger than others. Considering previous studies and a comparative approach to investigate the properties of 9 plants of the Apiaceae family, the present study determined the potential of these essential oils in terms of antimicrobial properties and effective doses. In this regard, the essential oils of 3 plants, *K. odoratissima*, *F. gummosa*, and *H. persicum*, with their specific chemical profiles, had a favorable potential in inhibiting the growth of various bacteria. The essential oil of *H. persicum* was more effective than other plants in inhibiting *C. albicans* and *A. flavus*. Thus, the essential oil of this plant can be a suitable option for medical applications to inhibit this human pathogen *C. albicans* and to inhibit fungal food contamination and storage loss of *A. flavus*. The essential oils of *C. cyminum* and *B. persicum* plants had very high antibacterial properties compared to others, hence, both plants are more emphatically recommended for industrial and medical applications. Also, the results of AHC and PCA analysis showed that the creation of mathematical models for predicting the biological properties of essential oils based on their constituents can be effective and it is suggested to pay more attention to this finding in the future.

## Data Availability

Raw data and derived data supporting the findings of this study are available from the corresponding author on request.
